# Surveillance of West Nile Virus in Tunisia: Evidence from Human and Entomological Investigation

**DOI:** 10.3390/v17121562

**Published:** 2025-11-29

**Authors:** Walid Barhoumi, Marwa Khedhiri, Youmna M’Ghirbi, Souha Bougatef, Henda Touzi, Adel Rhim, Hela Karray, Abdelhalim Trabelsi, Maha Mastouri, Naila Hannachi, Ali Bouattour, Henda Triki, Nissaf Bouafif Ben Alaya, Wasfi Fares

**Affiliations:** 1 National Observatory for New and Emerging Diseases, Ministry of Health, Tunis 1002, Tunisia; walidbarhoumi.ipt2009@yahoo.fr (W.B.); souhabg@yahoo.fr (S.B.); nissaf.bouafif@fmt.utm.tn (N.B.B.A.); 2 Laboratory of Virus, Vector and Host (LR20IPT02), Pasteur Institute of Tunis, University Tunis El Manar, Tunis 1002, Tunisia; marwa.kehidiri@gmail.com (M.K.); youmna.mghirbi@pasteur.utm.tn (Y.M.); touzihenda@yahoo.fr (H.T.); adel.rhim@yahoo.fr (A.R.); bouattourali74@yahoo.com (A.B.); henda.triki@pasteur.tn (H.T.); 3 Laboratory of Clinical Virology, WHO Reference Laboratory for Poliomyelitis and Measles in the Eastern Mediterranean Region, Pasteur Institute of Tunis, Tunis 1002, Tunisia; 4 Laboratory of Medical Entomology, Pasteur Institute of Tunis, Tunis 1002, Tunisia; 5 Laboratory of Microbiology, CHU Habib Bourguiba, Sfax 3027, Tunisia; hela.karray@rns.tn; 6 Laboratory of Microbiology, CHU Sahloul, Sousse 4054, Tunisia; abdelhalim.trabelsi@gmail.com; 7 Laboratory of Microbiology, CHU Fattouma Bourguiba, Monastir 5000, Tunisia; mastourimaha@yahoo.fr; 8 Laboratory of Microbiology, CHU Farhat Hached, Sousse 4000, Tunisia; naila.hannachi@rns.tn

**Keywords:** West Nile virus, *Culex pipiens*, *Culex perexiguus*, *Culex theileri*, *Aedes caspius*, vector surveillance, sublineage 1a, Tunisia

## Abstract

West Nile virus (WNV) is a mosquito-borne pathogen of the *Flaviviridae* family that poses recurring public health threats in Tunisia, where *Culex pipiens* is recognized as the primary vector. Identification of circulating strains in different mosquito species is essential for targeted prevention and control. Between November 2021 and October 2022, mosquitoes were collected at four high-risk sites, and human samples were obtained through the national meningitis surveillance program. Human serum, cerebrospinal fluid (CSF), and urine samples were tested for WNV-specific IgM and IgG antibodies using ELISA, and molecular diagnosis was performed using Real-time RT-PCR (RRT-PCR). Positive samples underwent sequencing for phylogenetic characterization. Serological investigation on human serum revealed the presence of IgM and/or IgG antibodies reactive to WNV antigens, which may indicate exposure to WNV or related flaviviruses. RNA of WNV was detected in 21 mosquito pools (10.19%) belonging to *Culex pipiens*, *Cx. perexiguus*, *Aedes caspius,* and *Ae. detritus*, as well as in three human cases. Phylogenetic analysis of positive human and mosquito samples showed that all detected WNV strains belonged to sublineage 1a. The concurrent detection of WNV in vectors and humans confirms active circulation in Tunisia and underscores the role of *Culex* spp. Mosquitoes in transmission. Sustained multidisciplinary surveillance integrating entomological and clinical data is critical for early detection, guiding control measures, and preventing future outbreaks in humans and animals.

## 1. Introduction

West Nile virus (WNV) is an emerging zoonotic arbovirus that belongs to the *Orthoflavivirus* genus, *Flaviviridae* family. It naturally circulates between birds and mosquitoes [1,2]. Currently, this arbovirus is the most widely distributed among encephalitic flaviviruses [3,4,5,6], making it a vector-borne pathogen of global importance [7]. Occasionally, a WNV-infected mosquito bites humans or other mammals, which are considered end-hosts [8,9,10,11]. The enzootic cycle is driven by continuous virus transmission to susceptible and/or infected bird species through adult female mosquito blood meals, leading to virus amplification. A wide range of mosquito species transmit West Nile virus [12]. *Culex* mosquitoes are reported to be the primary competent vectors of WNV [13,14,15]. The main vectors of WNV in Europe include *Cx. pipiens*, *Cx. modestus*, *Cx. perexiguus*, *Ae. caspius*, *Ae. vexans*, *Cx. torrentium*, *An. maculipennis*, and *Coquillettidia richiardii* [16,17,18,19]. WNV is endemic in Middle Eastern countries and is largely transmitted by *Cx. pipiens*, *Cx. perexiguus*, and *Ae. caspius* [20,21,22]. In Asia, the main vectors include *vishnui* complex, *Cx. fatigans*, *Cx. tritaeniorhynchus*, *Cx. bitaeniorhynchus*, *Cx. univittatus*, *Ae. albopictus*, and *Cx. Tritaeniorhynchus* [23]. In the Americas, the principal vectors are *Cx. restuans* and *Cx. salinarius* [24]. In Africa, the most important vectors are *Cx. univittatus*, *Cx. antennatus*, *Cx. pipiens*, and *Cx. Theileri* [25,26,27].

**Figure 1:**
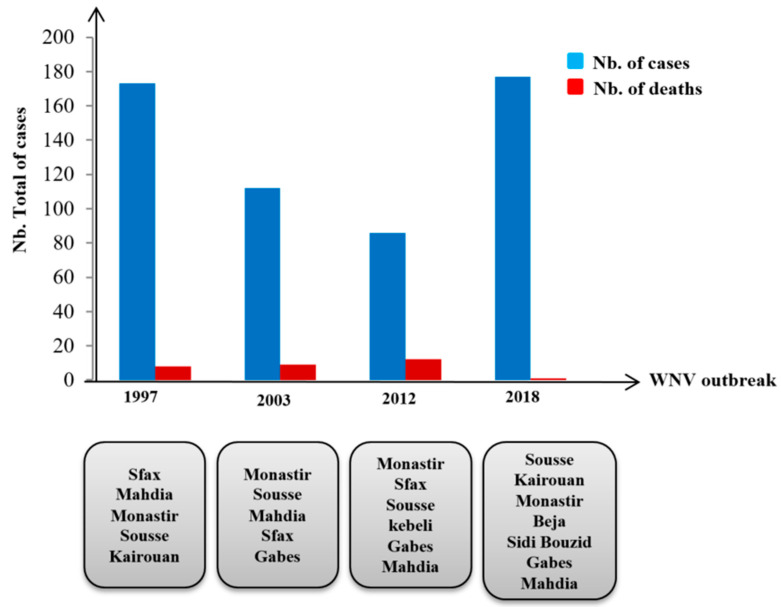
Neuroinvasive outbreaks in humans in TUNISIA.

In Tunisia, neuroinvasive outbreaks in humans were reported in 1997 (173 cases with eight deaths), in 2003 (21 cases and three deaths), and in 2012 (86 cases and 12 deaths) [28,29,30,31,32]. In 2018, an outbreak of encephalitis and meningitis was observed, and WNV infections were diagnosed by serology and molecular methods [33]. Sporadic cases were also recorded in 2007, 2010, and 2011 [34,35] (Figure 1). However, entomological data on WNV circulation in mosquito vectors remain limited.

Coastal regions of eastern Tunisia, mainly the governorates of Sousse and Monastir, including villages such as Sahline (35°46′ N, 10°46′ E) and Msaken (35°43′ N, 10°36′ E), have experienced four WNV outbreaks and are considered high-risk areas [36]. The locality of Ichkeul (37°10′ N, 9°43′ E), a coastal region in northern Tunisia, serves as a major wintering and stopover site for migratory birds. A previous study documented WNV circulation in horses in this area [37]. The governorate of Kairouan, particularly El Metbassta (35°46′ N, 10°07′ E), also recorded several human cases during the 2018 outbreak.

In recent years, environmental changes in Tunisia, such as rising temperatures, increased rainfall, and expanded irrigation, have created favorable conditions for the geographical spread of WNV, mirroring trends in other Mediterranean countries [38,39,40]. Human-induced habitat changes, including irrigation in arid and semi-arid areas, directly impact mosquito breeding and the distribution of ecological niches supporting virus transmission [41,42]. In Tunisia, despite repeated outbreaks of WNV, information on naturally infected mosquito vectors remains scarce, and most available studies have focused either on human or animal cases without integrating entomological and clinical surveillance in parallel. Moreover, since the 2018 outbreak, there has been no updated molecular and phylogenetic characterization of WNV strains in the country. These gaps limit our understanding of the ongoing circulation of WNV and the potential role of different mosquito species in its transmission.

In the present study, we aimed to address these gaps by conducting integrated One Health surveillance of WNV in Tunisia during the 2021–2022 transmission season. We combined serological and molecular testing of human samples with molecular analysis of mosquito pools. Mosquito species positive for WNV were identified. Furthermore, sequencing and phylogenetic analysis were performed to characterize the circulating WNV strains and compare them with previously reported Tunisian and regional strains.

## 2. Materials and Methods

### 2.1. Human Surveillance: Sample Collection, Serology and Molecular Testing

**Figure 2:**
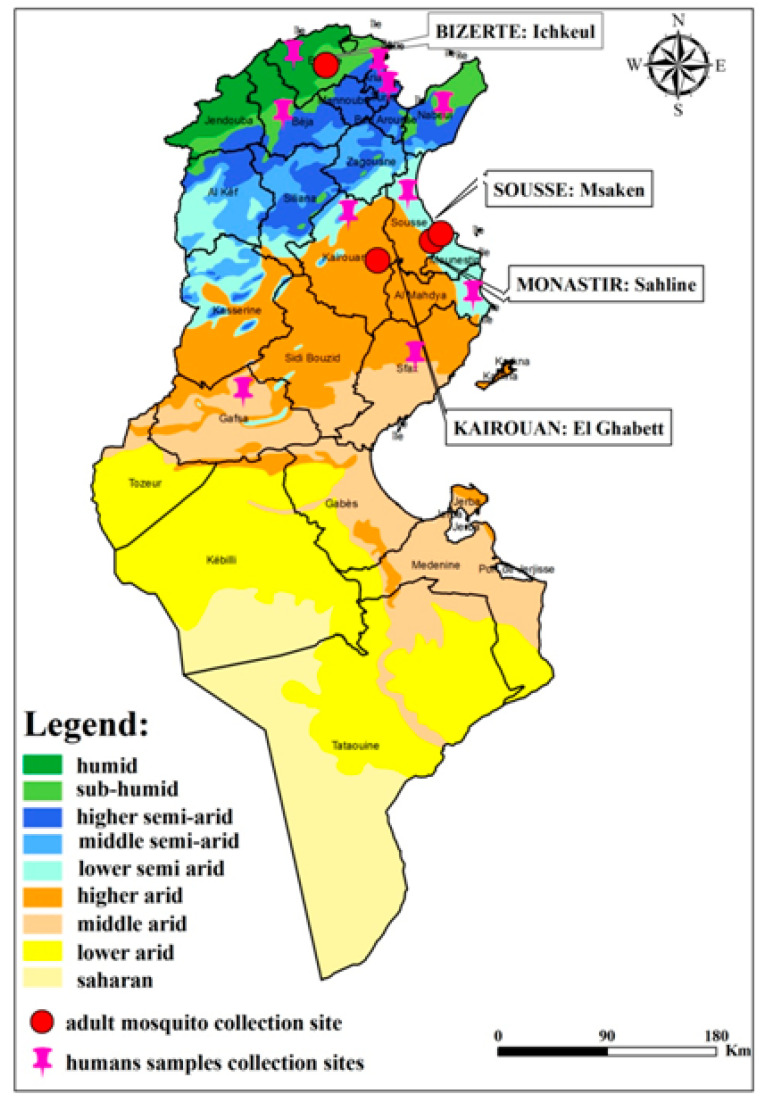
Geographical distribution of human and mosquito sampling sites in Tunisia, according to climatic and ecological conditions.

As part of the national monitoring program for human meningitis associated with WNV infections, 87 serum samples, 33 cerebrospinal fluid (CSF) samples, and 51 urine samples were collected during 2021 and 2022. According to the national case definition, patients were enrolled if they presented with clinical symptoms compatible with viral meningitis or meningoencephalitis including fever, headache, neck stiffness, altered consciousness, or other neurological manifestations in the absence of bacterial etiology. These suspected cases were reported through the national notifiable diseases system. The patients’ ages ranged from 5 to 78 years (median: 37 years). Samples were collected within 1–10 days after symptom onset, following standardized procedures of the national surveillance program. Blood samples were drawn into serum tubes, urine samples were collected in sterile containers, and CSF samples were obtained via lumbar puncture under aseptic conditions. All specimens were transported at 4 °C to the National Reference Laboratory of Clinical Virology at the Pasteur Institute of Tunis and processed within 24 h for serological and molecular testing. Clinical and demographic information was systematically recorded using a standardized case investigation form. The collected samples originated from ten different Tunisian governorates: Tunis, Bizerte, Nabeul, Sfax, Ariana, Sousse, Kairouan, Béja, Gafsa, and Mahdia (Figure 2). Clinical and demographic data were recorded using a standardized information sheet.

Serological testing for WNV-specific IgM and IgG antibodies was performed using commercial direct enzyme-linked immunosorbent assay (ELISA) kits (Euroimmun^®^, Lübeck, Germany), with percentages of sensitivity and specificity amounted to 94.4% and 99.8%, respectively. Serum samples were tested at a 1:101 dilution, and both known positive and negative control samples were included in each assay to validate its performance. The cut-off values for positive and negative results were determined based on the manufacturer’s recommended thresholds. Each assay plate included manufacturer-supplied positive, negative, and calibrator controls. According to the Euroimmun^®^ instructions, results were evaluated semi-quantitatively by calculating the ratio of the optical density (OD) of each sample to that of the calibrator. The OD values of the positive controls consistently ranged between [e.g., 1.8–2.2], well above the manufacturer’s cutoff (OD ratio > 1.1), while the negative controls yielded OD ratios between [e.g., 0.1–0.2], clearly below the cutoff (OD ratio < 0.8). According to the guidelines of the national program for WNV surveillance established since 2010, serum and urine samples are collected from all suspect cases. Case classification follows the World Health Organization and Tunisian Ministry of Health recommendations. A patient is considered a probable case when WNV-specific IgM antibodies are detected in serum or CSF, with or without IgG, in the absence of other etiologies. A confirmed case is defined by either detection of WNV RNA by real-time RT-PCR or demonstration of IgG seroconversion between acute and convalescent samples. During the study period, 6 patients with an initial IgM+/IgG− profile were resampled after 10–14 days and remained classified as probable cases due to absence of seroconversion and negative molecular results.

Viral RNA was extracted from 140 µL of CSF, Urine and mosquito homogenate using the QIAamp Viral RNA Mini Kit (QIAGEN^®^, Hilden, Germany). Molecular testing was performed on 5 µL of extracted RNA from human samples (CSF and urine) and mosquito homogenates using the SuperScript™ III OneStep rRT-PCR System with Platinum™ Taq DNA Polymerase (Invitrogen, Carlsbad, CA, USA) in the Applied Biosystems™ 7500 real-time PCR machine. Adopted molecular RRT-PCR method target a 92-nucleotide sequence within the highly conserved 3′ non-coding region of the WNV genome [43]. To ensure reliable results, known positive and negative WNV RNA samples were included in each assay as controls. Ct values of less than 35 were considered indicative of a positive result, with values greater than 35 deemed negative, as determined by previous studies and optimized during assay validation.

Positive samples were subsequently amplified by heminested RT-PCR, targeting a 408-nucleotide-long fragment in the junction between the C-terminal portion of the C gene and the N-terminal part of the prM gene. Amplification was performed in an end point thermocycler using the SuperScript II One-Step RT-PCR System with Platinum Taq DNA Polymerase kit (Invitrogen, Carlsbad, CA, USA) by reverse transcription PCR as previously described [33].

### 2.2. Entomological Surveillance

Entomological surveillance was conducted from November 2021 to October 2022 in four Tunisian governorates: Bizerte, Sousse, Monastir, and Kairouan (Figure 2). Study sites were selected based on epidemiological data, particularly in areas with known WNV circulation or wetlands considered at risk for WNV introduction through migratory birds. Mosquito collection sites were identified based on historical data on WNV circulation in humans and birds, and during periods of high transmission risk with important vector densities.

Adult mosquitoes were collected using CDC miniature light traps (John W. Hock Co., Gainesville, FL, USA) placed in animal shelters and were run overnight. In total, 168 traps were deployed in the 4 sites, approximately 1.5 m above the ground (52 CDC in Monastir, 36 in Sousse, 49 in Bizerte and 31 in Kairouan). The traps were transferred on dry ice to the laboratory and kept in the freezer for 15 min to immobilize the mosquitoes. Live mosquitoes were first identified morphologically to the species level using standard identification key [44]. Specimens were then pooled (up to 15 mosquitoes per pool) according to sex, species, collection site, and date. Pools were homogenized in 500 µL of Minimum Essentiel Medium (MEM) supplemented with antibiotic solution using the automated Tissue Lyser LT (QIAGEN^®^, Hidden, Germany) with glass beads at a frequency of 50 Hz for 5 min. Pools were and stored at −80 °C until RNA extraction. Based on virological results in mosquitoes, we can estimate the Minimum Infection rate (MIR). The MIR could be calculated as: MIR = Number of Positive Pool/Total Number of Mosquitoes Tested scale.

### 2.3. Sequencing and Phylogenetic Analysis

Positive samples identified by RRT-PCR-were further amplified using heminested reverse transcription PCR to obtain a 408-nucleotide fragment spanning the C-PrM junction, covering the C-terminal region of the C gene and the N-terminal region of the PrM gene, as previously described [33].

PCR products were purified using the ExoSAP kit (Thermo Fisher Scientific, CA, USA) and sequenced bidirectionally with the same PCR primers using the BigDye Terminator v3.1 cycle sequencing kit (Thermo Fisher Scientific, San Diego, CA, USA). Consensus sequences were used for phylogenetic analysis using the MEGA v.X software [45]. The phylogenetic tree was constructed from 96 WNV sequences retrieved from GenBank, covering wide geographical regions including six WNV lineages (1 to 5, and 7). Tree topology was inferred using the Neighbor-Joining method with the Kimura 2-parameter model and 1000 bootstrap replicates. The Usutu virus (USUV, NC006551) was used as an outgroup. Genetic distances were calculated using the p-distance method.

## 3. Results

### 3.1. Human Surveillance (Serological and Molecular Results)

A total of 87 serum samples were collected over the study period, including 25 in 2021 and 62 in 2022. Various serological profiles for WNV were identified. While the ELISA results suggest the presence of antibodies reactive to WNV antigens, this assay cannot distinguish between WNV and other closely related flaviviruses such as Usutu virus (USUV), which is known to circulate in Tunisia. Therefore, the observed reactivity should be interpreted with caution and does not confirm WNV-specific infection. Future studies will include virus neutralization tests to resolve this cross-reactivity.

In 2021, 4 samples tested positive for both IgM and IgG (IgM+/IgG+), 2 were IgM−/IgG+, and 19 were negative for both markers (IgM−/IgG−); no sample showed an isolated IgM+ response. In 2022, 7 samples were IgM+/IgG+, 6 were IgM+/IgG−, 1 was IgM−/IgG+, and 48 showed no serological evidence of WNV infection (IgM−/IgG−). The patients exhibiting an isolated IgM response were sampled within the first week after symptom onset, which likely explains the absence of detectable IgG due to the early phase of infection.

WNV infection etiology was confirmed by RRT-PCR in three human cases, among 51 tested urine sample collected in 2022. These cases presented clinical symptoms of meningoencephalitis and originated from the governorates of Mahdia, Sousse, Kairouan, Béja, and Tunis. No CSF or serum samples or urine samples tested positive for WNV RNA by RRT-PCR. Results of human serological and molecular findings are summarized in Table 1.

### 3.2. Entomological Surveillance (Species Identification and RRT-PCR Results)

A total of 1408 female mosquitoes were collected from November 2021 to October 2022 and pooled into 206 pools, with up to 15 specimens per pool. Morphological identification revealed four mosquito genera (*Culex*, *Aedes*, *Culiseta*, and *Anopheles*) and eight species. The identified species were: 661 females *Cx. pipiens* in 92 pools, 249 females *Cx. perexiguus* in 35 pools, 104 females *Cx. theileri* in 16 pools, 356 females *Ae. caspius* in 46 pools, 34 females *Ae. detritus* in 13 pools, 2 females *Cs. longiareolata*, 1 females *An. algeriensis*, and 1 females *An. labranchiae.*

Of the 206 mosquito pools tested, 21 were positive according to RRT-PCR, with a minimum infection rate (MIR) of 1.49%. Positive mosquito pools were collected from animal shelters located in peri-domestic areas (Table 2). The 21 positive pools belonged to four mosquito species: *Cx. pipiens* (10 pools), *Cx. perexiguus* (6 pools), *Ae. caspius* (4 pools), and *Ae. detritus* (1 pool).

Most of the WNV-positive pools had high Ct values (>35) in RRT-PCR, indicating a low viral load in the mosquito vectors. Only one pool of *Cx. perexiguus*, with a Ct value of 28, tested positive by heminested RT-PCR.

### 3.3. Sequencing and Phylogenetic Analysis Results

**Figure 3:**
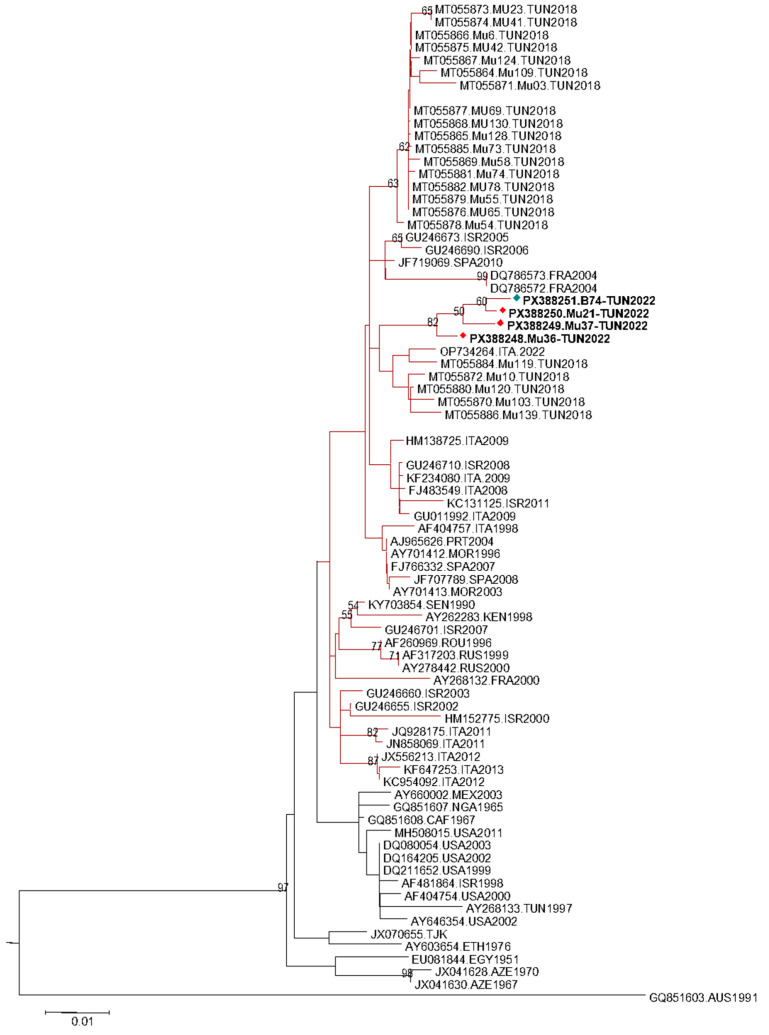
West Nile virus lineage 1 phylogenetic subtree: Phylogenetic tree was generated using MEGA v.X software. Tree topology was inferred using the Neighbor-Joining method with the Kimura 2-parameter model and 1000 bootstrap replicates. The Usutu virus (USUV, NC006551) was used as an outgroup. Human generated sequences are marked by a red diamond while the sequence originating from mosquito is marked in green. For clarity, only representative sequences of sub-lineage 1a are displayed. Phylogenetic branches of the Mediterranean subtype’s representative sequences are highlighted in red.

The generated partial sequences, including three from human samples (MU21, MU36, MU37) and one from mosquito (B74), were submitted to GenBank database under accession numbers PX388248-PX388251. Sequences were confirmed as WNV by BLAST analysis (https://blast.ncbi.nlm.nih.gov/ accessed on 5 November 2025) and were phylogenetically classified as WNV sublineage 1a (Figure 3). Newly generated sequences, obtained during the same transmission season, clustered together within the Mediterranean subtype in a single phylogenetic cluster supported by a bootstrap value of 82. They were closely related to WNV strains previously detected in Tunisia in 2018 and to one sequence from Italy identified in 2022 in bird host.

## 4. Discussion

West Nile Virus (WNV) continues to pose significant public health challenges, particularly in regions with environmental conditions favorable to its transmission. In this study, we explored the circulation of WNV in Tunisia through serological and molecular investigations involving both human and mosquito populations. WNV infections have been reported in humans and equines across various regions, including Europe, Australia, Asia, the Middle East, the USA, and Africa [33,38,46,47,48,49,50]. Tunisia has experienced several WNV outbreaks, with the most recent documented between August and November 2018 [33].

Our findings indicate a variable seroprevalence of WNV antibodies in human samples over the study period. Specifically, out of 25 serum samples collected in 2021, 4 were positive for IgM antibodies. In 2022, among 62 urine samples tested by real-time RT-PCR, none were positive for WNV RNA. The relatively low number of positive samples in both investigated years suggests the absence of an epidemic situation. However, it is important to note that the ELISA results were not confirmed by virus neutralization tests (VNTs), which are considered the gold standard for WNV sero-diagnosis. It is also important to note that the ELISA test used in this study may detect antibodies generated in response to other flaviviruses, particularly Usutu virus (USUV), which has been documented as enzootic in Tunisia [51]. Therefore, the detected reactivity reflects probable exposure to WNV or antigenically related flaviviruses, rather than confirmed WNV infection. This limitation should be taken into account, particularly given the possibility of cross-reactions with other closely related circulating *orthoflaviviruses*. This finding is in agreement with earlier Tunisian studies that reported sporadic WNV antibody detection in humans during inter-epidemic periods [30,35], as well as outbreak investigations documenting WNV encephalitis cases in 2003 and 2012 [27,52]. It is also consistent with other studies from the Mediterranean region where WNV circulation is often sporadic although other countries such as Italy, Greece, and more recently Spain, have experienced recurrent epidemic seasons [53]. Our results therefore provide updated evidence that WNV continues to circulate at low levels in Tunisia between major outbreaks, reinforcing the need for sustained human serological surveillance.

Entomological surveillance identified several mosquito species naturally infected with WNV, particularly *Cx. pipiens* with an infection rate (IR) of 1.51%, previously identified as the principal WNV vector in Tunisia [42,54,55,56,57]. While *Cx. pipiens* is already known to play a role in WNV transmission in Tunisia, we report here for the first time natural WNV infection in *Cx. perexiguus*, notably with a high MIR, as well as in *Ae. caspius* and *Ae. detritus*, all recognized or potential vectors of WNV. However, the most frequently infected species in our mosquito pools was *Cx. perexiguus*. This species is recognized as a WNV vector in the Middle East, Europe [58], and Turkey [59]. Its natural infection with WNV was reported for the first time in Northern Africa in Algeria [60] and more recently in Tunisia [61,62]. Given its abundance in the four study areas and an infection rate of 2.4%, *Cx. perexiguus* should be considered a key species in WNV surveillance efforts.

Interestingly, *Ae. caspius* pools positive for WNV (*n* = 4) were detected in Kairouan, a region known to have experienced several human WNV cases during the 2018 epidemic. This species thrives in sebkha environments, which are saline, sparsely vegetated wetlands—an ideal ecological niche for *Ae. caspius* [44]. Such areas also promote interaction between mosquitoes and migratory birds, facilitating virus transmission. *Ae. caspius* has been reported as a WNV vector in Italy [15]. The natural infection observed in our study may result from recent blood meals on infected birds.

In addition, we detected WNV RNA in a single pool of *Ae. detritus* collected in Sousse. However, one positive pool is insufficient to definitively incriminate this species as a WNV vector. Overall, WNV infection was detected in four mosquito species: *Cx. pipiens*, *Cx. perexiguus*, *Ae. caspius*, and *Ae. detritus*, with respective infection rates of 1.51%, 2.4%,1.12%, and 2.94%. These results confirm the major role of *Cx. pipiens* as the principal vector of WNV and suggest that *Cx. perexiguus* and *Ae. caspius* may also play significant roles in transmission in Tunisia.

The analysis of adult mosquito collections from Sousse (Msaken), Monastir (Sahline), Bizerte (Ichkeul), and Kairouan (El Metbassta) showed notable variations in species abundance and infection rates. The geographical distribution and infection data suggest that urban settings like Sousse and Monastir may present different transmission dynamics compared to rural areas such as Kairouan and Bizerte. Urban areas may support higher mosquito densities, potentially enhancing virus transmission. Conversely, fewer positive pools in rural areas might indicate that while competent vectors are present, environmental conditions such as habitat and land use may limit transmission intensity.

Understanding WNV epidemiology in Tunisia requires a broader surveillance approach. Antibodies against WNV have been detected in dromedaries, birds, and equids, indicating past exposure and suggesting their involvement in the virus’s enzootic cycle [32,34,61,62]. Recent study indicates that ruminants such as sheep, goats, and camels could serve as sentinel species, especially in rural areas, to support early detection and targeted control efforts [63]. Molecular investigations confirmed the presence of WNV RNA in both human and mosquito samples. Previous Tunisian studies have reported WNV RNA either in human cases, such as during the 2018 neuroinvasive outbreak [33], or in mosquito vectors, as documented in 2014 [42]. Our study extends these observations by providing integrated molecular evidence of WNV circulation in both humans and mosquitoes during the same transmission seasons (2021–2022). Phylogenetic analysis, based on amplified partial genome region, further revealed that all sequenced strains belong to sublineage 1a, which is predominant in the Mediterranean Basin as well as in Tunisia, where previous studies have consistently identified lineage 1a as the dominant circulating strain [33,61]. This finding is also consistent with previous studies identifying lineage 1 as the dominant strain in Europe, Africa, and the Middle East [61,64]. Interestingly, the strains detected in 2022 formed a distinct monophyletic group closely related to strains from Tunisia (2018) [33] and Italy (2022) [65]. The close homology with the Tunisian 2018 strains suggests the possibility of viral maintenance within the country, and the similarity with the 2022 strain isolated in *Pica pica* (Eurasian magpie) from the Veneto region of Italy further supports the contribution of this resident species to WNV maintenance within its geographical range, acting as a potential donor and/or recipient of viral strains between resident and migratory avifauna (medium- and long-distance migrants) [66]. Overall, the integration of molecular and phylogenetic data adds originality to our study and delivers updated evidence on the circulation dynamics of WNV in Tunisia. Similar patterns have been reported in the region, supporting the role of migratory birds in WNV dispersal [8].

The clustering of Tunisian strains into separate phylogenetic subclusters reflects the genetic diversity of circulating WNV and suggests either multiple introductions or local viral evolution. Our findings are consistent with those from Italy and Greece, where lineage 1a also predominates with notable genetic variability [18]. However, the relatively high Ct values observed in our mosquito pools suggest low viral loads at the time of collection, possibly indicating reduced vector competence or transmission intensity. This contrasts with findings from southern Spain, where high viral loads correlated with intense local transmission and larger outbreaks [67]. Thus, the presence of WNV in several mosquito species in our study, combined with lower viral loads, may indicate broad but moderate virus circulation, a pattern observed in areas with stable endemicity [68].

Our phylogenetic data are consistent with previously published observations regarding the presence of WNV lineage 1a in both mosquito and human populations. This sublineage has been implicated in numerous outbreaks across Europe, North Africa, and the Middle East, and appears well-adapted to transmission in these regions [3]. The close genetic proximity between Tunisian and Italian 2022 strains underlines the need for international cooperation in WNV surveillance and control. Molecular surveillance, as demonstrated here, is essential for monitoring virus evolution and guiding public health interventions.

## 5. Conclusions

This study underscores the importance of integrated surveillance to monitor WNV circulation in Tunisia. We report WNV infection in *Cx. pipiens*, *Cx. perexiguus*, *Ae. caspius*, and *Ae. detritus* across ecologically distinct regions of the country. In addition to confirming the role of *Cx. pipiens* and *Cx. perexiguus*, our results highlight *Ae. caspius*, and *Ae. detritus* as newly identified naturally infected species in Tunisia. The genetic diversity of WNV strains further emphasizes the need for continued molecular monitoring to detect viral evolution and new introductions. Moving forward, enhanced vector control strategies, particularly in high-risk areas, will be crucial to reduce transmission and prevent future outbreaks.

## Figures and Tables

**Table 1:** Summary of serological and molecular results of human samples collected in Tunisia (2021–2022).

Year	Sample Type	No. Tested	IgM+/IgG+	IgM+/IgG−	IgM−/IgG+	IgM−/IgG−	RRT-PCR Positive (Sample Type)
2021	Serum	25	4	0	2	19	0 (all tested negative)
2022	Serum	62	7	6	1	48	0 (all tested negative)
2021–2022	CSF	33	–	–	–	–	0
2021–2022	Urine	51	–	–	–	–	3 (all in 2022)
**Total**	All samples	87 (serum), 33 (CSF), 51 (urine)	11	6	3	67	3 (urine, 2022)

**Table 2:** The distribution of mosquito species and pools according to the sampling sites in Tunisia.

Gouvernorat	Site (GPS Coordinates)	Biotope	Mosquito Species	Nb. of Tested Mosquitoes	Nb. of Tested Pools	RRT-PCRPositive WNV Pools	MIR (%)
Sousse	**Msaken** **(35°43′ N, 10°36′ E)**	Urban	*Cx. pipiens*	307	38	4	1.3
			*Cx. perexiguus*	108	15	3	2.77
			*Cx. theileri*	15	3	-	
			*Ae. caspius*	54	7	-	
			*Ae. detritus*	2	04	-	
			*Cs. longiareolata*	1	01	-	
Monastir	**Sahline** **(35°46′ N, 10°46′ E)**	Urban	*Cx. pipiens*	57	10	1	1.75
			*Cx. perexiguus*	37	5	-	
			*Cx. theileri*	01	02	-	
			*Ae. caspius*	69	12	-	
			*Ae. detritus*	09	04	1	11.11
Bizerte	**Ichkeul** **(37°10′ N, 9°43′ E)**	Rural	*Cx. pipiens*	192	31	2	1.04
			*Cx. perexiguus*	26	05	-	
			*Cx. theileri*	01	01	-	
			*Ae. caspius*	08	03	-	
			*Ae. detritus*	18	04	-	
			*Cs. longiareolata*	01	01	-	
			*An. labranchiae*	01	01	-	
Kairouan	**Metbasta** **(35°46′ N, 10°07′ E)**	Rural	*Cx. pipiens*	105	13	3	2.85
			*Cx. perexiguus*	78	10	3	3.84
			*Cx. theileri*	87	10	-	
			*Ae. caspius*	225	24	4	1.77
			*Ae. detritus*	05	01	-	
			*An. algeriensis*	01	01	-	
**Total**				**1408**	**206**	**21**	**1.5**

## Data Availability

The data that support the findings of this study are available from the corresponding author upon reasonable request.

## Abbreviations

The following abbreviations are used in this manuscript:

WNV	West Nile virus
*Cx.*	*Culex*
*Ae.*	*Aedes*
*An.*	*Anopheles*
*Cs.*	*Culiseta*
RNA	Ribonucleic acid
RRT-PCR	Real time reverse transcription polymerase chain reaction
PCR	Polymerase chain reaction
CSF	Cerebrospinal fluid
ELISA	Enzyme-linked immunosorbent assay
IR	Infection rate
MIR	Minimum infection rate
Ct	Cycle threshold
BLAST	Basic Local Alignment Search Tool
USUV	Usutu virus
